# Circadian Rhythmic Characteristics in Men With Substance Use Disorder Under Treatment. Influence of Age of Onset of Substance Use and Duration of Abstinence

**DOI:** 10.3389/fpsyt.2018.00373

**Published:** 2018-08-17

**Authors:** Maria del Mar Capella, Antonio Martinez-Nicolas, Ana Adan

**Affiliations:** ^1^Department of Clinical Psychology and Psychobiology, Faculty of Psychology, University of Barcelona, Barcelona, Spain; ^2^Chronobiology Lab, Department of Physiology, College of Biology, University of Murcia, IUIE, IMIB-Arrixaca, Murcia, Spain; ^3^Ciber Fragilidad y Envejecimiento Saludable (CIBERFES), Madrid, Spain; ^4^Institute of Neurosciences, University of Barcelona, Barcelona, Spain

**Keywords:** abstinence, circadian rhythm, chronotype, distal skin temperature, onset of substance use, sleep-wake schedules, substance use disorder

## Abstract

There is evidence of the reciprocal influence between the alteration of circadian rhythms and Substance Use Disorders (SUD), and part of the success of the SUD treatment lays in the patient's rhythmic recovery. We aim to elucidate the effect of the SUD treatment in circadian rhythmicity considering, for the first time, the age of onset of substance use (OSU) and duration of abstinence. We registered the sleep-wake schedules, the chronotype and the distal skin temperature of 114 SUD patients with at least 3 months of abstinence, considering whether they had begun consumption at age 16 or earlier (OSU ≤ 16, *n* = 56) or at 17 or later (OSU ≥ 17, *n* = 58), and duration of abstinence as short (SA: 3 to 5 months, *n* = 38), medium (MA: 6 to 9 months, *n* = 35) or long (LA: more than 9 months, *n* = 41). Moreover, we compared the patients' distal skin temperature pattern with a similar sample of healthy controls (HC, *n* = 103). SUD patients showed a morningness tendency and higher night values, amplitude and stability, a better adjustment to the cosine model and lower minimum temperature and circadianity index in the distal skin temperature rhythm, in contrast to the HC group. The OSU ≥ 17 and LA groups showed a more robust distal skin temperature pattern, as well as milder clinical characteristics when compared to the OSU ≤ 16 and SA groups, respectively. The circadian disturbances associated to substance consumption seem to improve with treatment, although the age of OSU and the duration of abstinence are modulating variables. Our results highlight the need to include chronobiological strategies that boost circadian rhythmicity both in SUD prevention and rehabilitation programs. The measurement of distal skin temperature rhythm, a simple and reliable procedure, could be considered an indicator of response to treatment in SUD patients.

## Introduction

During adolescence there is a higher risk to begin substance consumption (1, 2), and an early age of onset of substance use (OSU) is associated to the future development of Substance Use Disorders (SUD) (3) and to more severe clinical characteristics (4–8). Therefore, it is crucial to improve substance consumption prevention programs, as well as to consider the age of OSU and its clinical implications in SUD treatments. Chronobiology is here an area of high interest thanks to the evidence collected in the last decades.

Biological rhythms are essential to survival, and their alteration has been consistently linked to a wide range of health problems (9, 10), including SUD. Circadian rhythms (lasting around 24 h) deserve special attention given their relevance in both work and clinical performance. They are endogenously generated by the body's biological clock, located in the suprachiasmatic nucleus of the hypothalamus, although they are synchronized with the environmental rhythm of light-darkness (11, 12). Several functions, both physiological (body temperature, hormone secretion, wake-sleep cycle, etc.) and psychological (mood, cognitive performance, etc.), show an evident circadian rhythmicity (10, 13–15).

Some parameters, such as body temperature or melatonin secretion, are considered biological markers of circadian rhythmicity (16, 17). In recent years, there is a growing interest to measure distal skin temperature, since it is easy to record and reflects internal temporal order reliably (18–21). Distal skin temperature has been validated for sleep-wake detection (22), and it can be used instead of dim-light melatonin onset to predict the internal phase (21), since it remains after demasking procedures (20). Its usefulness has been proven in very different populations, among others, babies, and older people (23), mild cognitive impairment (24), sleep-disordered breathing (25), or in patients with metabolic syndrome (26).

Different studies show that substance consumption alters the expression of circadian rhythmicity, and this can last for months after the onset of abstinence (27, 28).

The most frequent alterations are a decrease in the amplitude and a phase delay in circadian functions, seen both in body temperature and in melatonin secretion (29–31). In this regard, the genes regulating the circadian system seem to be involved in the activity of the reward system in response to substance use (32–34), and it is suggested that alterations in its activity contribute to the vulnerability of developing a SUD (34–36). Moreover, substance consumption may modify the genetic expression with more or less permanent changes, depending on the severity of consumption and the subject's vulnerability (37).

Individuals show circadian rhythm differences depending on their chronotype or circadian typology (morning-, neither-, and evening-type). This is an individual difference, usually assessed with self-informed questionnaires (38), and which several recent studies have shown to be a crucial health variable. Thus, the evening chronotype is considered a risk factor for substance consumption both for youth and adults (36, 39, 40). This has been linked to obtaining a higher reinforcement with consumption (37, 41, 42), and with a greater social jet-lag or desynchronization between the biological and the social clocks (43, 44). Moreover, evening-type subjects show more personality traits associated to substance use (45, 46), worse academic and work adjustment (40, 47), less ability to cope with stress (48), and worse quality of life (49).

Several studies have highlighted the importance of the age of onset of substance use on the consequences of those who develop a SUD. When compared to people who begin consumption at 17 or later, those who begin at 16 or earlier have a lower premorbid intelligence quotient (7, 50), a worse neuropsychological performance (51, 52), maladaptive treatment-coping strategies (8), and a lesser volume of gray brain matter (53). This cut-off age has been based on neurodevelopmental characteristics, such as a possible dysfunction in the dopaminergic and endocannabinoid systems. These systems are key to prefrontal function, have a final peak of cortical changes at about age 15, and are almost completely defined toward the end of puberty (36, 54). Bearing in mind that dopamine has been established as a modulator of the circadian system (36, 55), it is necessary to explore the possible rhythmic differences in SUD patients considering this cut-off age of onset of consumption. SUD treatment requires an integral, long-lasting approach, where the first year of treatment is considered as a phase of early remission (56), where it is useful to prescribe sleep schedules and stable, morning-type activities to patients. However, we are not aware of any previous studies addressing the influence of age of onset of consumption and duration of abstinence in the rhythmic circadian expression of SUD patients undergoing treatment.

Our work has two aims. The first is to examine the differences in the clinical characteristics of male patients under treatment for SUD, and after the detox phase, depending on whether they initiated substance use at age 16 or earlier (OSU ≤ 16) or at age 17 or later (OSU ≥ 17), and the duration of abstinence (short, medium or long). The second is to explore the differences in the circadian rhythmicity of distal skin temperature in SUD patients, according to the age of OSU and duration of abstinence, compared with a similar sample of healthy controls. We also measure circadian typology and sleep-wake schedules for SUD patients.

## Materials and methods

### Study design and participants

In a cross-sectional study design, participants were recruited from March 2014 to July 2017. A total of 156 patients were referred to our study by their treating psychiatrist or psychologist. All of them were under SUD treatment in specialized healthcare resources (ambulatory or residential in a therapeutic community) from Barcelona. Forty-one patients were excluded due to refuse to participate in the study or dropout of it (*n* = 11), psychiatric comorbidity (*n* = 2), drug relapse (*n* = 5), treatment dropout (*n* = 8), refuse temperature monitoring (*n* = 6), or invalid temperature recording (*n* = 9). Thus, 114 participants satisfied our inclusion/exclusion criteria and SUD disorder with cocaine, alcohol, cannabis or opioids as primary drug of dependence. The inclusion criteria were: (a) aged 18–55; (b) current or past diagnosis of SUD according to the criteria in the Diagnostic and Statistical Manual of Mental Disorders, 4thEdition Text Revised (DSM-IV-TR) (57); and (c) in abstinence for at least 3 months (excluding caffeine or nicotine consumption), to ensure the overcoming of withdrawal symptoms and minimum adherence to treatment, confirmed by urinalysis. The exclusion criteria were: (a) presence of any other medical problems which could interfere in the assessment (such as sensorial deficits or neurological injury); and (b) presence of a comorbid axis I mental disorder confirmed by a diagnostic interview according to DSM-IV-TR criteria. All patients were male, given the high prevalence of this gender in SUD (2) and to avoid bias on the results due to sex differences (23, 41). After collecting the data, patients were assigned to groups according to the age of OSU and the duration of abstinence. To study the effect of the age of OSU, they were assigned to two groups: age of OSU at 16 or earlier (OSU ≤ 16; *n* = 56), or at 17 or later (OSU ≥ 17; *n* = 58). The groups based on duration of abstinence were for short abstinence (SA; *n* = 38) from 3 to 5 months, for medium abstinence (MA; *n* = 35) from 6 to 9 months, and for long abstinence (LA; *n* = 41) when more than 9 months.

Healthy controls volunteers (HC; *n* = 103) were recruited from the community of Murcia and Barcelona with exclusion criteria similar than those in the SUD groups, with the added requirement that they must not present any current or past DSM-IV-TR (57) diagnosis.

This study was approved by the ethics committees of the University of Barcelona for the SUD and HC participants, and also by the University of Murcia in the case of the HC. The study was conducted in accordance with the ethical principles of the declaration of Helsinki (58) and the international ethical standards of chronobiological research (59). Study participants provided written informed consent for participation and were not compensated.

### Sociodemographic and clinical measures

Sociodemographic data (age, years of education, marital, and economic status) and clinical (presence of substance use and psychiatric pathology in family history, suicidal attempts, consumption pattern, type of drugs used, age of OSU, duration of drug use, type of treatment, current medication, abstinence period, past treatment for SUD, previous relapses, and number of daily cigarettes and caffeine beverages) were collected by means of a structured interview designed specifically for our study, and the Structural Clinical Interview for the DSM-IV Axis I Disorders (SCID-I) (60). This information was confirmed through the patients' treating psychiatrist and the medical history of the centers' databases.

The Clinical Global Impression questionnaire (CGI) (61) was applied as a subjective measure of clinical severity. The Spanish version (62) of the Drug Abuse Screening Test (DAST-20) (63) was administered to assess the severity of the SUD. This test provides a total score from 0 to 20 (0 indicating no addiction, 1–5 low, 6–10 intermediate, 11–15 substantial, and 16–20 severe). Furthermore, the Seasonal Pattern Assessment Questionnaire (SPAQ) (64) in its Spanish version (65) was used to assess seasonality of mood variations. The SPAQ assigns a Global Seasonality Score (GSS) determined by responses to six indices of seasonality (sleep length, mood, social activity, weight, appetite, and energy level) ranging from 0 to 24, and also considers the degree in which seasonal changes are a problem (no, mild, moderate, marked, severe, or disabling). Three categories are established: no seasonal affective disorder [GSS < 10], subsyndrome of seasonal affective disorder [9 < GSS < 12 and seasonal variations being perceived as a problem, or GSS > 11 but seasonal changes being of minor degree], and seasonal affective disorder [GSS > 11 and seasonal variations considered minimum as a moderate problem].

### Circadian typology and sleep-wake data

The Composite Scale of Morningness (CSM) (66) in its Spanish version (67) was administered to SUD patients as a measure of circadian typology. This test is composed of 13 items with Likert-scale answers, with a total score from 13 to 55. In the Spanish version, the scores for the circadian typologies were 13–25 for the evening-type, 26–36 for the neither-type, and 37–55 for the morning-type. We also collected sleep-wake data through a structured interview designed specifically for our study: total time spent sleeping, wake-up time, bedtime, presence and duration of nap.

### Distal skin temperature measurement

Distal skin temperature was registered every 10 min for 48 h using the Thermochron iButton® DS1921H device (Maxim Integrated Products, Sunnyvale, California, USA), which had an accuracy of ±1°C at 0.125°C (19). In order to reduce the potential masking effect generated by the higher activity of the dominant hand, the sensor was placed on the wrist of the non-dominant hand over the radial artery (18). Participants were encouraged to maintain their usual lifestyles during temperature measurements.

### Statistical analyses

The temperature data were analyzed through the Circadianware™ version 7.1.1 software (68). The Cosinor analyses (parametric) were applied to calculate the maximum and minimum temperature, mesor, amplitude, acrophase, Rayleigh vector, and percentage of variance explained by the cosine wave (%V), as well as the Fourier analysis with the first 12 harmonics. Furthermore, the circadianity index was calculated in a similar way to that described in Batinga et al. (23). A nonparametric analysis was also performed, as previously described by Witting et al. (69) and Martinez-Nicolas et al. (70), that provided the interdaily stability (IS), intradaily variability (IV), relative amplitude (RA), maximum mean temperature in 5 consecutive hours (M5) and its respective timing (TM5), minimum mean temperature in 2 consecutive hours (L2) and its timing (TL2), minimum mean temperature in 10 consecutive hours (L10) and its timing (TL10). Furthermore, the mean temperature values of four main phases according to local time were calculated, as explained in Batinga et al. (23): morning decrease (08:00–15:00 h), afternoon secondary peak (15:00–18:00 h), evening decrease (18:00–23:00 h), and night plateau (23:00–08:00 h).

Descriptive statistics and frequencies were calculated for all groups considered. Differences between groups in the sociodemographic and clinical variables were explored with the Mann-Whitney U test (*U*) or with the Chi Square test (χ^2^) for categorical variables. If the quantitative data fulfilled the necessary conditions, the Student's *t*-test (*t*) or the one-way analysis of variance (ANOVA) were used. Otherwise, the non-parametric Mann-Whitney *U*-test (*U*) or the Kruskal-Wallis test were used instead. The internal consistency for the CSM was calculated with the Cronbach's alpha coefficient. Differences in CSM scores were assessed using analysis of covariance (ANCOVA), while the sleep-wake schedules, distal skin temperature parametric (cosinor) and non-parametric data were assessed with three multivariate analyses of covariance (MANCOVA). Age was considered as covariate in all cases, since it could be a confounding factor. These comparisons were performed first attending to the diagnostic (HC or SUD groups), second considering the HC and the age of OSU (OSU ≤ 16 or OSU ≥ 17) groups, and third attending to the HC and duration of abstinence (SA, MA, or LA) groups, except for the sleep-wake schedules and CSM, since no data were recorded for the HC participants. Additional analyses were performed considering the type of treatment (residential or ambulatory), to assess whether this was an indicator of differences in some circadian measure. The Bonferroni's test was applied in all analyses to reduce the occurrence of a type I error. The effect size was calculated with the partial Eta squared (η^2^_*p*_), assuming a value of 0.01 as low, of 0.06 as moderate and of 0.14 as high (71). Statistical analyses were performed using the Statistical Package for the Social Sciences (SPSS; version 22.0), considering bilateral statistical significance with an established type I error at 5% (*p* < 0.05).

## Results

### Sociodemographic and clinical data

Attending to the sociodemographic data (Table 1), the HC and SUD groups did not differ in age. Nevertheless, although most of the participants had completed the Spanish compulsory education (from 6 to 16 years, grades 1–10), the SUD patients had a lower mean in years of education (*p* < 0.001). Marital status showed higher rates of singles in the SUD group compared to higher rates of married persons in the HC group (*p* < 0.001), as well as higher rates of unemployed SUD patients compared to higher active workers in the HC group (*p* < 0.001). The age of OSU and abstinence groups, with no significant age differences between them, had similar results to those obtained by the SUD group compared with the HC group. There were differences in the years of education between the HC and the age of OSU groups (*p* < 0.001), as well as in the abstinence groups (*p* < 0.001). The post-hoc analyses showed that the OSU ≤ 16 (*p* < 0.001), SA (*p* < 0.001), MA (*p* = 0.019), and LA (*p* < 0.001) patients had fewer years of education than the HC participants. Moreover, the age of OSU (*p* < 0.001) and the abstinence (*p* < 0.001) groups showed lower rates of married and employed persons (*p* < 0.001) than the HC group.

Table 1Sociodemographic data for all groups.

**Table:** 

	**HC (*n* = 103)**	**Total SUD (*n* = 114)**	**Statistical contrasts**
Age (yrs)	37.68 ± 1.11	35.26 ± 0.71	*t* = −1.877
Years of education	12.50 ± 0.19	10.28 ± 0.22	*U* = 1494^***^
Marital status			χ^2^ = 30.484^***^
Single	24.6%	53.5%	
Separate/Divorced	9.8%	19.20%	
Married	44.3%	14.9%	
Stable partner	19.7%	12.3%	
Economic status			χ^2^ = 103.839^***^
Active	93.9%	25.4%	
Unemployed	1.0%	31.6%	
No income	0%	21.1%	
Disability pension	5.1%	14.9%	
Sick leave	0%	7%

**Table:** 

	**HC (*****n*** = **103)**	**OSU ≤ 16 (*****n*** = **56)**	**OSU ≥ 17 (*****n*** = **58)**	**Statistical contrasts**	**HC (*****n*** = **103)**	**SA (*****n*** = **38)**	**MA (*****n*** = **35)**	**LA (*****n*** = **41)**	**Statistical contrasts**
Age (yrs)	37.68 ± 1.11	34.11 ± 1.17	36.40 ± 0.79	*F* = 1.034	37.68 ± 1.11	34.50 ± 1.21	33.94 ± 1.02	37.10 ± 1.33	*F* = 0.976
Years of education	12.50 ± 0.19	10.10 ± 0.32	10.47 ± 0.31	*F* = 23.070^***^	12.50 ± 0.19	9.89 ± 0.32	10.58 ± 0.47	9.85 ± 0.33	*F* = 19.112^***^
Marital status				χ^2^ = 28.649^***^					χ^2^ = 122.075^***^
Single	24.6%	50%	56.9%		24.6%	57.9%	65.7%	39%	
Separate/Divorced	9.8%	21.4%	17.2%		9.8%	26.4%	5.7%	24.4%	
Married	44.3%	14.3%	15.5%		44.3%	7.9%	11.4%	24.4%	
Stable partner	19.7%	14.3%	10.3%		19.7%	7.9%	17.1%	12.2%	
Economic status				χ^2^ = 108.343^***^					χ^2^ = 41.789^***^
Active	93.9%	16.1%	34.5%		93.9%	15.8%	22.9%	36.6%	
Unemployed	1.0%	32.1%	31%		1.0%	26.3%	37.1%	31.7%	
No income	0%	25%	17.2%		0%	31.6%	14.3%	17.1%	
Disability pension	5.1%	19.6%	10.3%		5.1%	18.4%	11.4%	14.6%	
Sick leave	0%	7.1%	6.9%		0%	7.9%	14.3%	0%	

*HC, healthy controls; SUD, substance use disorder; yrs, years; OSU ≤ 16, onset of substance use at age 16 or earlier; OSU ≥ 17, onset of substance use at age 17 or later; SA, short abstinence; MA, medium abstinence; LA, long abstinence*.

*** *p < 0.001*.

The analyses of the clinical variables indicated significant differences in the age of the OSU groups, being more frequent in the OSU ≤ 16 group to have polydrug use (*p* = 0.014), residential rather than ambulatory treatment (*p* = 0.010), and longer duration of drug use (*p* < 0.001). Furthermore, the groups showed differences in the type of substances used. In the OSU ≤ 16 group, there were higher rates of cannabis (*p* = 0.001) and hallucinogens consumption (*p* = 0.033). In the total SUD sample, as well as in both groups, the substances more frequently used were cocaine (88.6%), alcohol (71.1%), and cannabis (42.1%). No differences between the OSU groups were found in the other clinical characteristics studied. Regarding the abstinence groups, higher percentage of relatives with SUD was found in the MA group (*p* = 0.026) in contrast with the SA and LA groups. There were also significant differences in type of treatment, with a higher frequency of patients in residential treatment in the SA group with respect to the MA and LA groups (*p* < 0.001). Moreover, the CGI showed main effects (*p* = 0.012), being the LA group significantly better compared to the SA group (*p* = 0.031). There were no statistically significant differences in the daily consumption of medication in the pharmacologically treated patients between age of OSU groups or among abstinence groups (see Table 2). Moreover, the opioid agonists prescription did not show statistical differences for age of OSU groups (13% for OSU ≤ 16 and 15.5% for OSU ≥ 17; χ^2^ = 0.149, *p* = 0.699) or abstinence groups (18.4% for SA, 11.8% for MA, and 12.5% for LA; χ^2^ = 0.811, *p* = 0.667).

**Table 2:** Clinical data for the whole SUD sample and all their subgroups.

**Clinical data**	**Total SUD (*n* = 114)**	**OSU ≤ 16 (*n* = 56)**	**OSU ≥ 17 (*n* = 58)**	**Statistical contrasts**	**SA (*n* = 38)**	**MA (*n* = 35)**	**LA (*n* = 41)**	**Statistical contrasts**
Relatives with SUD	18.4%	23.2%	13.8%	χ^2^ = 1.683	18.4%	31.4%	7.3%	χ^2^ = 7.305^*^
Relatives with others psychiatric disorder	24.6%	25%	24.1%	χ^2^ = 0.011	31.6%	14.3%	26.8%	χ^2^ = 3.118
Number of suicidal attempts	0.25 ± 0.06	0.29 ± 0.78	0.22 ± 0.56	*U* = 1616.50	0.29 ± 0.12	0.17 ± 0.57	0.29 ± 0.11	*F* = 0.376
Consumption pattern								
One substance	28.9%	21.4%	36.2%	χ^2^ = 3.025	42.1%	17.1%	26.8%	χ^2^ = 5.659
Two substances	32.5%	28.6%	36.2%	χ^2^ = 0.758	21.1%	37.1%	39%	χ^2^ = 3.412
Polydrug use	38.6%	50%	27.6%	χ^2^ = 6.039^*^	36.8%	45.7%	34.1%	χ^2^ = 1.140
Substances used^a^								
Cocaine	88.6%	83.9%	93.1%	χ^2^ = 2.374	84.2%	93.4%	87.6%	χ^2^ = 2.101
Alcohol	71.1%	75%	67.2%	χ^2^ = 0.834	57.9%	80%	75.6%	χ^2^ = 4.975
Cannabis	42.1%	57.1%	27.6%	χ^2^ = 10.212^***^	36.8%	48.6%	41.5%	χ^2^ = 1.039
Hallucinogens	15.8%	23.2%	8.6%	χ^2^ = 4.564^**^	15.8%	14.3%	17.1%	χ^2^ = 0.110
Opioids	16.7%	19.6%	13.8%	χ^2^ = 0.702	18.4%	17.1%	14.6%	χ^2^ = 0.212
Sedatives	1.8%	1.8%	1.7%	χ^2^ = 0.001	2.6%	0%	2.4%	χ^2^ = 0.906
Age of OSU (yrs)	18.67 ± 0.49	15.32 ± 0.39	21.90 ± 0.65	*U* = 73.50^**^	17.34 ± 0.71	19.71 ± 0.88	19.01 ± 0.92	*F* = 2.008
Duration of drug use (yrs)	16.35 ± 0.81	18.10 ± 1.18	14.66 ± 1.08	*t* = 2.162^***^	16.74 ± 1.21	14.30 ± 1.42	17.73 ± 1.50	*F* = 1.567
Typology of treatment regimen				χ^2^ = 6.634^*^				χ^2^ = 17.021^***^
Residential	63.2%	75%	51.7%		89.5%	51.4%	48.8%	
Ambulatory	36.8%	25%	48.3%		10.5%	48.6%	51.2%	
Daily number of medication	0.52 ± 0.08	0.64 ± 0.14	0.40 ± 0.09	*U* = 1483	0.58 ± 0.17	0.54 ± 0.16	0.44 ± 0.11	*F* = 0.264
Months of abstinence	8.18 ± 0.45	7.64 ± 0.62	8.69 ± 0.66	*U* = 1404.50	4.11 ± 0.11	7.11 ± 0.17	13.12 ± 0.73	*F* = 100.643^***^
Past treatment for SUD	49%	45.3%	53.2%	χ^2^ = 0.623	53.3%	56.3%	39.5%	χ^2^ = 2.278
Number of previous relapses								
None	57%	53.6%	60.3%	χ^2^ = 0.533	52.6%	54.3%	63.4%	χ^2^ = 1.089
One	16.7%	14.3%	19%	χ^2^ = 0.449	15.8%	20%	14.6%	χ^2^ = 0.423
Two	10.5%	14.3%	6.9%	χ^2^ = 1.652	13.2%	11.4%	7.3%	χ^2^ = 0.758
Three or more	15.8%	17.9%	13.8%	χ^2^ = 0.354	18.4%	14.3%	14.6%	χ^2^ = 0.299
DAST-20. Direct Score	12.41 ± 0.46	12.76 ± 0.56	12.03 ± 0.75	*t* = 0.788	12.41 ± 0.79	12.16 ± 0.78	12.70 ± 0.84	*F* = 0.114
Addiction severity (DAST-20)				χ^2^ = 0.676				χ^2^ = 0.813
Low/Intermediate	32.9%	35.1%	30.3%		27.3%	36%	34.8%	
Substantial	40.0%	35.1%	45.5%		45.5%	40%	34.8%	
Severe	27.1%	29.7%	24.2%		27.3%	24%	30.4%	
CGI. Direct Score	2.45 ± 0.11	2.57 ± 0.17	2.33 ± 0.15	*U* = 1428	2.29 ± 0.20	2.39 ± 0.14	2.80 ± 0.20	*F* = 4.636^*^
Daily number of cigarettes	12.50 ± 0.78	12.54 ± 1.11	12.47 ± 1.11	*U* = 1593	11.82 ± 1.28	11.09 ± 1.26	14.34 ± 1.44	*F* = 1.654
Daily beverages with caffeine	1.88 ± 0.15	1.86 ± 0.23	1.90 ± 0.20	*t* = 0.045	1.82 ± 0.29	1.63 ± 0.22	2.15 ± 0.27	*F* = 0.979
SPAQ. Direct Score	5.76 ± 0.46	6.02 ± 0.70	5.52 ± 0.61	*t* = 0.541	6.05 ± 0.82	5.60 ± 0.89	5.63 ± 0.72	*F* = 0.097
Seasonal Affective Disorder (SPAQ)	15%	16.4%	13.8%	χ^2^ = 0.146	13.2%	17.6%	15.1%	χ^2^ = 0.201

*SUD, Substance use disorder; OSU ≤ 16, onset of substance use at age 16 or earlier; OSU ≥ 17, onset of substance use at age 17 or later; SA, short abstinence; MA, medium abstinence; LA, long abstinence; OSU, Onset of Substance Use; yrs, years; DAST-20, Drug Abuse Screening Test; CGI, Clinical Global Impression; SPAQ, Seasonal Pattern Assessment Questionnaire*.

a *Percentages will not equal 100 as each participant may take more than one substance of abuse*.

* *p < 0.05*,

** *p < 0.01*,

*** *p < 0.001*.

### Circadian typology and sleep-wake characteristics

Cronbach's alpha coefficient of internal reliability for the CSM was adequate for the total SUD sample studied (0.826). Attending to the age of OSU groups, the ANCOVA analysis did not show significant differences in the CSM total score. It is worth mentioning that we found a high percentage of the morning-type in the SUD patients, especially in the OSU ≥ 17 group (70.7%). The MANCOVA analyses for the sleep-wake schedules (total time spent sleeping, wake-up time, bedtime, presence, and duration of nap) also did not yield any significant differences. Similarly, no differences were found in the CSM total score, the circadian typology and the sleep-wake schedules parameters in the duration of abstinence groups (Table 3).

**Table 3:** Circadian typology and sleep-wake data for the whole SUD sample and all their subgroups.

**Circadian typology and sleep-wake data**	**Total SUD (*n* = 114)**	**OSU ≤ 16 (*n* = 56)**	**OSU ≥ 17 (*n* = 58)**	**Statistical contrasts**	**SA (*n* = 38)**	**MA (*n* = 35)**	**LA (*n* = 41)**	**Statistical contrasts**
CSM. Direct Score	37.96 ± 6.57	36.81 ± 6.79	39.02 ± 6.22	*F* = 2.702	38.03 ± 6.17	37.89 ± 6.38	38.13 ± 7.20	*F* = 0.012
Circadian typology (CSM)				χ^2^ = 2.386				χ^2^ = 1.301
Morning-type	64.3%	57.4%	70.7%		62.2%	65.7%	65%	
Neither-type	30.4%	35.2%	25.9%		35.1%	28.6%	27.5%	
Evening-type	5.4%	7.4%	3.4%		2.7%	5.7%	7.5%	
Total time spent sleeping (hrs)	8.01 ± 0.91	8.09 ± 0.91	7.92 ± 0.84	*F* = 0.790	8.07 ± 0.99	8.13 ± 0.70	7.87 ± 0.90	*F* = 0.912
Wake-up time^a^	06:53 ± 01:42	06:39 ± 02:08	07:07 ± 01:07	*F* = 1.073	06:33 ± 01:32	07:18 ± 01:29	06:52 ± 02:16	*F* = 1.208
Bedtime^a^	23:20 ± 01:17	23:12± 01:38	23:28 ± 00:59	*F* = 1.405	23:22 ± 01:15	23:26 ± 00:57	23:14 ± 1:42	*F* = 0.302
Nap duration (min)	18.09 ± 30.58	20.19 ± 35.97	16.14 ± 24.69	*F* = 0.459	20.14 ± 30.02	17.21 ± 34.26	19.02 ± 23.09	*F* = 0.038
Presence of nap	30.4%	35.2%	25.9%	χ^2^ = 0.221	31.6%	34.3%	31.7%	χ^2^ = 0.077

*SUD, Substance use disorder; OSU ≤ 16, onset of substance use at age 16 or earlier; OSU ≥ 17, onset of substance use at age 17 or later; SA, short abstinence; MA, medium abstinence; LA, long abstinence; CSM, Composite Scale of Morningness; hrs, hours; min, minutes*.

a *Data are expressed in hours and minutes*.

In the additional analyses carried out considering the type of treatment (residential or ambulatory) as an independent variable no significant differences between groups were found regarding the CSM scores or sleep-wake schedules (*p* > 0.193; in all cases).

### Distal skin temperature

The circadian rhythmic characteristics are shown in Table 4 for the HC and the SUD groups. The MANCOVA analyses revealed several significant differences between the HC and SUD groups. SUD patients were characterized by higher values in minimum temperature (*p* = 0.012), amplitude (*p* = 0.001), Rayleigh vector (*p* = 0.001), %V (*p* = 0.001), first harmonic power (*p* = 0.001) and accumulated power after 12 harmonics (*p* = 0.001), IS (*p* = 0.001), lower values in the circadianity index (*p* = 0.001), and an advanced acrophase (*p* = 0.019) in contrast to the HC group.

**Table 4:** Distal skin rhythmic variables for the HC, SUD and age of OSU groups.

	**HC (*n* = 103)**	**Total SUD (*n* = 114)**	***F***	**η^2^*_*p*_***	**HC (*n* = 103)**	**OSU ≤ 16 (*n* = 56)**	**OSU ≥ 17 (*n* = 58)**	***F***	**η^2^*_*p*_***	**Significant contrasts**
**COSINOR DATA**										
Maximum temp	36.07 ± 0.46	36.11 ± 0.59	0.280		36.07 ± 0.46	36.02 ± 0.56	36.19 ± 0.61	1.484		
Minimum temp	30.39 ± 1.66	30.87 ± 1.68	6.430^*^	0.029	30.39 ± 1.66	31.12 ± 1.52	30.63 ± 1.79	5.671^**^	0.054	HC < OSU ≤ 16
Mesor	33.60 ± 0.63	33.63 ± 0.86	0.192		33.60 ± 0.63	33.71 ± 0.86	33.55 ± 0.87	1.291		
Amplitude	0.79 ± 0.44	1.07 ± 0.71	10.844^**^	0.048	0.79 ± 0.44	0.87 ± 0.58	1.26 ± 0.77	11.001^***^	0.099	HC, OSU ≤ 16 < OSU ≥ 17
Acrophase^a^	02:06 ± 01:39	00:41 ± 03:17	11.886^**^	0.053	02:06 ± 01:39	00:48 ± 03:09	00:35 ± 03:25	5.097^**^	0.048	OSU ≤ 16, OSU ≥ 17 < HC
Rayleigh vector	0.75 ± 0.18	0.91 ± 0.19	35.155^***^	0.175	0.75 ± 0.18	0.89 ± 0.20	0.92 ± 0.19	18.981^***^	0.160	HC < OSU ≤ 16, OSU ≥ 17
%V	18.16 ± 11.35	26.63 ± 18.02	17.416^***^	0.078	18.16 ± 11.35	23.09 ± 16.22	29.90 ± 19.12	11.424^***^	0.103	HC, OSU ≤ 16 < OSU ≥ 17
1st HP	0.40 ± 0.42	0.79 ± 1.04	11.849^**^	0.053	0.40 ± 0.42	0.55 ± 0.75	1.03 ± 1.23	11.839^***^	0.106	HC, OSU ≤ 16 < OSU ≥ 17
12th HAP	0.69 ± 0.62	1.60 ± 1.61	27.165^***^	0.113	0.69 ± 0.62	1.29 ± 1.46	1.90 ± 1.70	17.048^***^	0.146	HC < OSU ≤ 16 < OSU ≥ 17
Circadianity index	50.60 ± 20.58	42.12 ± 21.85	8.014^**^	0.036	50.60 ± 20.58	37.87 ± 21.50	46.24 ± 21.81	6.212^**^	0.055	OSU ≤ 16 < HC
**NON-PARAMETRIC DATA**										
Interdaily Stability (IS)	0.40 ± 0.15	0.75 ± 0.16	26.407^***^	0.552	0.40 ± 0.15	0.72 ± 0.15	0.77 ± 0.17	20.364^***^	0.256	HC < OSU ≤ 16, OSU ≥ 17
Intradaily Variability (IV)	0.23 ± 0.10	0.24 ± 0.10	0.215		0.23 ± 0.10	0.26 ± 0.17	0.22 ± 0.11	1.984		
Relative Amplitude (RA)	0.02 ± 0.01	0.03 ± 0.02	9.635^**^	0.043	0.02 ± 0.01	0.03 ± 0.02	0.04 ± 0.02	9.497^***^	0.087	HC, OSU ≤ 16 < OSU ≥ 17
MD temp	34.41 ± 0.58	34.47 ± 0.72	3.642		34.41 ± 0.58	34.46 ± 0.63	34.49 ± 0.80	1.829		
ASP temp	33.10 ± 0.94	32.92 ± 1.29	1.321		33.10 ± 0.94	33.03 ± 1.35	32.82 ± 1.22	1.148		
ED temp	33.48 ± 1.09	33.46 ± 1.31	0.003		33.48 ± 1.09	33.69 ± 1.19	33.24 ± 1.38	2.228		
NP temp	33.28 ± 1.02	33.26 ± 1.34	0.033		33.28 ± 1.02	33.35 ± 1.35	33.17 ± 1.32	0.606		
M5	34.58 ± 0.49	34.88 ± 0.84	9.690^**^	0.043	34.58 ± 0.49	34.90 ± 0.82	34.86 ± 0.87	6.464^**^	0.061	HC < OSU ≤ 16, OSU ≥ 17
TM5^a^	02:39 ± 02:08	01:51 ± 03:01	3.523		02:39 ± 02:08	01:47 ± 02:12	01:55 ± 02:53	1.530		
L2	31.99 ± 1.31	31.64 ± 1.79	2.024		31.99 ± 1.31	31.89 ± 1.67	31.40 ± 1.88	2.669		
TL2^a^	20:20 ± 05:21	20:52 ± 06:16	0.140		20:20 ± 05:21	21:33 ± 05:18	20:14 ± 06:59	0.322		
L10	32.99 ± 0.86	32.85 ± 1.29	0.663		32.99 ± 0.86	33.06 ± 1.21	32.65 ± 1.34	3.122		
TL10^a^	14:02 ± 03:19	13:15 ± 02:59	3.603		14:02 ± 03:19	13:16 ± 02:35	13:14 ± 03:23	1.523		

*HC, healthy controls; SUD, Substance use disorder; OSU, onset of substance use; η^2^_p_, partial eta square; OSU ≤ 16, onset of substance use at age 16 or earlier; OSU ≥ 17, onset of substance use at age 17 or later temp temperature; %V, percentage of variance explained by the cosine wave; HP, harmonic power; HAP, harmonic accumulated power; MD, morning decrease; ASP, afternoon secondary peak; ED, evening decrease; NP, night plateau; M5, maximum mean temperature in 5 consecutive hours; TM5, time when the maximum mean temperature in 5 consecutive hours was reached; L2, minimum mean temperature in 2 consecutive hours; TL2, time when the minimum temperature in 2 consecutive hours was reached; L10, minimum mean temperature in 10 consecutive hours; TL10, time when the minimum temperature in 10 consecutive hours was reached*.

a *Data are expressed in hours and minutes*.

* *p < 0.05*,

** *p < 0.01*,

*** *p < 0.001*.

**Figure 1:**
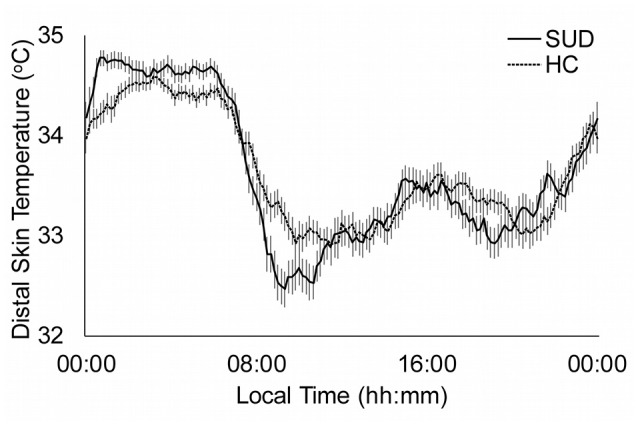
Distal skin temperature mean daily patterns for substance use disorder patients (SUD; continuous line, n: 114) and healthy controls (HC; dotted line, *n* = 103). All data are expressed as mean ± standard error of the mean.

The distal skin temperature functions for the HC and total SUD sample are presented in Figure 1. The SUD patients showed a pattern with higher and more stable values at night, in comparison to the HC group, as seen in the differences in the M5 (*p* = 0.002), and lower and more variable during the day. The rise in M5 in the SUD patients is related to the higher RA in the HC (*p* = 0.002).

The comparisons between the HC and the age of OSU groups are showed in Table 4. The two age of OSU groups had an advanced acrophase and higher values in the Rayleigh vector, IS and M5 than the HC group (*p* < 0.033, in all cases). The values of OSU ≥ 17 group were higher than the HC and OSU ≤ 16 groups in several cosinor and non-parametric data: amplitude, %V, first harmonic power and relative amplitude (*p* < 0.039, in all cases). The accumulated power after 12 harmonics was higher in the OSU ≥ 17 group in contrast to the values of HC and OSU ≤ 16 groups (*p* < 0.029), and also higher in the OSU ≤ 16 group than in the HC group (*p* = 0.031). The OSU ≤ 16 group showed higher values in minimum temperature (*p* = 0.009) and lower circadianity index (*p* = 0.002) with respect to the HC group, without differences with the OSU ≥ 17 group.

**Figure 2:**
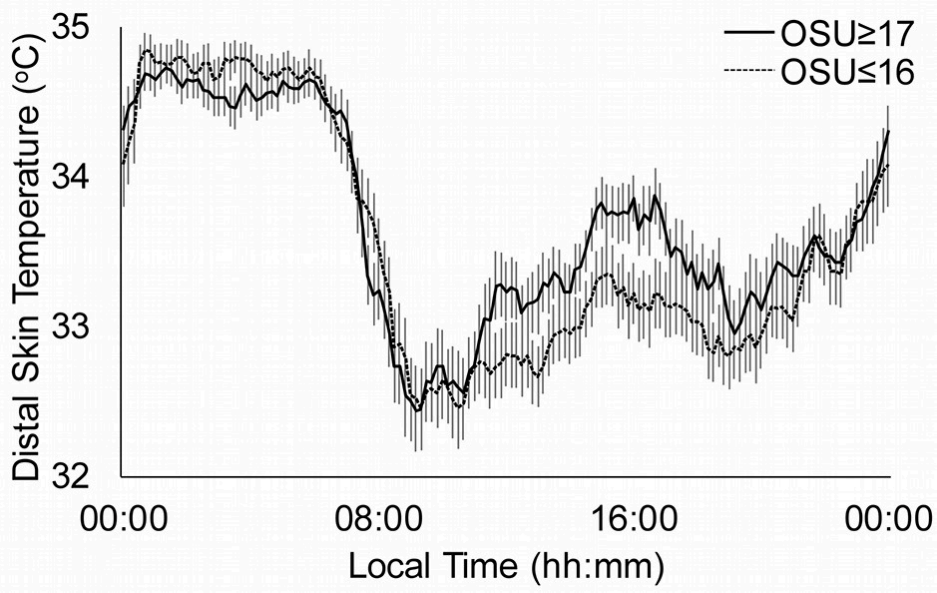
Distal skin temperature mean daily patterns for substance use disorders according to the age of Onset of Substance Use (OSU). Continuous line represents OSU ≥ 17 (*n* = 58) and dotted line OSU ≤ 16 (*n* = 56). All data are expressed as mean ± standard error of the mean.

Figure 2 shows the distal skin temperature functions for the OSU groups. The temperature values during the day (L10) were higher in the OSU ≤ 16 group, although the contrast with the OSU ≥ 17 group was not significant. However, there were significant differences in relative amplitude (*p* = 0.034), which was higher in the OSU ≥ 17 group.

On the other hand, significant statistical differences for several distal skin temperature variables were found between the HC and the abstinence groups (Table 5). The three abstinence groups were characterized by higher values in the Rayleigh vector (*p* < 0.004), accumulated power after 12 harmonics (*p* < 0.041), and IS (*p* < 0.045) in contrast to the HC group. We also found higher values of the MA and LA groups, compared with the HC and SA groups, on the amplitude (*p* < 0.002), first harmonic power (*p* < 0.032), and relative amplitude (*p* < 0.004). The MA and LA groups showed higher values in the M5 (*p* < 0.015) with respect to the HC group. Only the LA group showed higher values in %V (*p* < 0.001), in contrast to the HC group. The SA group presented a more advanced acrophase than the HC group (*p* < 0.001), and lower values in TL10 than the LA group (*p* < 0.049). Furthermore, the SA and MA groups showed lower values in the circadianity index (*p* = 0.017) with respect to the HC group. Finally, the LA patients had lower values in minimum temperature (*p* = 0.041) in contrast to the SA patients, and lower IV than the SA, and MA patients (*p* < 0.047).

**Table 5:** Distal skin circadian rhythmic variables for the HC and duration of abstinence groups.

	**HC (*n* = 103)**	**SA (*n* = 38)**	**MA (*n* = 35)**	**LA (*n* = 41)**	***F***	**η^2^*_*p*_***	**Significant contrasts**
**COSINOR DATA**							
Maximum temp	36.07 ± 0.46	36.07 ± 0.58	36.07 ± 0.56	36.20 ± 0.67	0.590		
Minimum temp	30.39 ± 1.66	31.30 ± 1.42	30.97 ± 1.84	30.43 ± 1.57	4.934^**^	0.069	LA, HC < SA
Mesor	33.60 ± 0.63	33.79 ± 0.84	33.70 ± 0.94	33.49 ± 0.77	1.471		
Amplitude	0.79 ± 0.44	0.84 ± 0.68	1.06 ± 0.68	1.33 ± 0.69	8.290^***^	0.111	HC, SA < LA
Acrophase^a^	02:06 ± 01:39	23:47 ± 04:02	01:23 ± 03:21	01:24 ± 04:16	6.047^**^	0.084	SA < HC
Rayleigh vector	0.75 ± 0.18	0.86 ± 0.25	0.91 ± 0.18	0.93 ± 0.15	13.093^***^	0.165	HC < SA, MA, LA
%V	18.16 ± 11.35	22.74 ± 17.82	25.72 ± 18.60	31.84 ± 17.42	7.748^***^	0.105	HC < LA
1st HP	0.40 ± 0.42	0.58 ± 1.12	0.78 ± 0.91	1.11 ± 1.11	7.211^***^	0.098	HC, SA < LA
12th HAP	0.69 ± 0.62	1.19 ± 1.44	1.57 ± 1.45	2.03 ± 1.77	12.730^***^	0.161	HC < SA, MA, LA
Circadianity index	50.60 ± 20.58	38.60 ± 22.95	37.85 ± 21.79	49.30 ± 19.35	5.137^**^	0.068	SA, MA < HC
**NON-PARAMETRIC DATA**							
Interdaily Stability (IS)	0.40 ± 0.15	0.70 ± 0.19	0.75 ± 0.16	0.75 ± 0.13	28.121^***^	0.541	HC < SA, MA, LA
Intradaily Variability (IV)	0.23 ± 0.10	0.26 ± 0.13	0.29 ± 0.19	0.18 ± 0.8	3.420^*^	0.049	LA < SA, MA
Relative Amplitude (RA)	0.02 ± 0.01	0.03 ± 0.02	0.03 ± 0.02	0.04 ± 0.02	7.909^***^	0.107	HC, SA < LA
MD temp	34.41 ± 0.58	34.44 ± 0.75	34.51 ± 0.74	34.48 ± 0.69	1.280		
ASP temp	33.10 ± 0.94	33.05 ± 1.32	32.96 ± 1.38	32.77 ± 1.19	0.853		
ED temp	33.48 ± 1.09	33.77 ± 1.21	33.49 ± 1.42	33.15 ± 1.25	1.948		
NP temp	33.28 ± 1.02	33.58 ± 1.05	33.08 ± 1.62	33.10 ± 1.28	1.677		
M5	34.58 ± 0.49	34.70 ± 0.96	35.02 ± 0.54	35.09 ± 0.89	6.507^***^	0.089	HC < MA, LA
TM5^a^	02:39 ± 02:08	01:09 ± 03:00	02:04 ± 03:51	02:39 ± 03:22	2.436		
L2	31.99 ± 1.31	32.03 ± 1.63	31.46 ± 2.23	31.42 ± 1.46	2.010		
TL2^a^	20:20 ± 05:21	22:12 ± 06:26	20:03 ± 05:49	20:13 ± 06:01	0.529		
L10	32.99 ± 0.86	33.10 ± 1.14	32.90 ± 1.28	32.53 ± 1.24	2.249		
TL10^a^	14:02 ± 03:19	12:46 ± 04:26	13:20 ± 02:03	14:26 ± 03:54	2.728^*^	0.040	SA < LA

*HC, healthy controls; SA, short abstinence; MA, medium abstinence; LA, long abstinence; η^2^_p_, partial eta square; temp, temperature; %V, percentage of variance explained by the cosine wave; HP, harmonic power; HAP, harmonic accumulated power; MD, morning decrease; ASP, afternoon secondary peak; ED, evening decrease; NP, night plateau; M5, maximum mean temperature in 5 consecutive hours; TM5, time when the maximum mean temperature in 5 consecutive hours was reached; L2, minimum mean temperature in 2 consecutive hours; TL2, time when the minimum temperature in 2 consecutive hours was reached; L10, minimum mean temperature in 10 consecutive hours; TL10, time when the minimum temperature in 10 consecutive hours was reached*.

a *Data are expressed in hours and minutes*.

* *p < 0.05*,

** *p < 0.01*,

*** *p < 0.001*.

**Figure 3:**
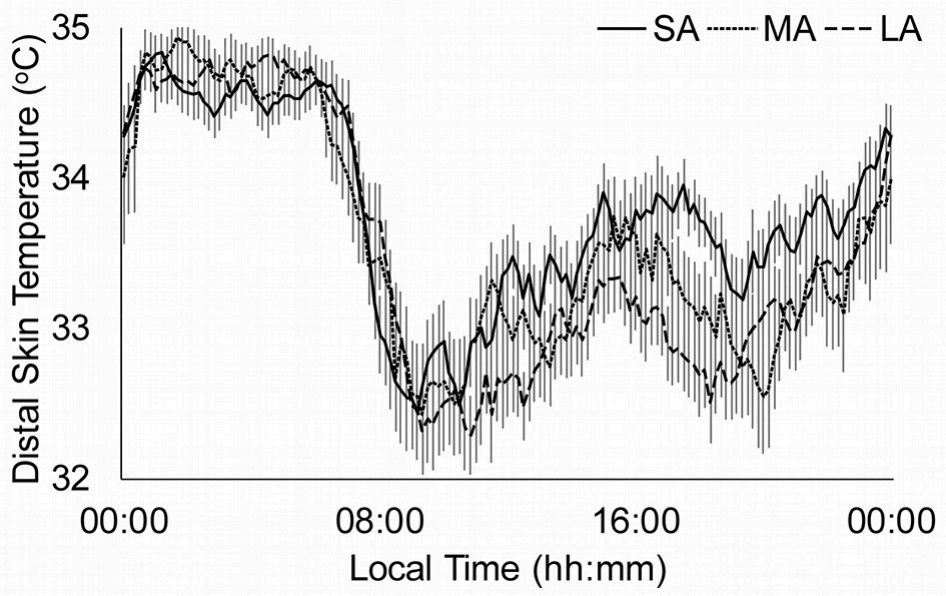
Distal skin temperature mean daily patterns for duration of abstinence. Continuous line represents short abstinence (SA, *n* = 38), dotted line medium abstinence (MA, *n* = 35) and discontinued line long abstinence (LA, *n* = 41). All data are expressed as mean ± standard error of the mean.

Figure 3 shows the distal skin temperature functions for the abstinence groups. There is a tendency to obtain higher values in the M5 and lower values in the L10 as the duration of abstinence increases. This is based in the significant differences found in the RA between the SA and LA groups. Moreover, there is also a phase delay in the TL10 and TM5 as the duration of abstinence increases.

In the additional analyses performed considering the type of treatment (residential or ambulatory) only a significant difference was found in acrophase [*F*_(1, 112)_ = 7.223; *p* = 0.008; η^2^_*p*_ = 0.061], being advanced in the ambulatory group.

## Discussion

This study intended to clarify, for the first time, the possible existence of clinical and circadian (sleep-wake schedules, circadian typology, and distal skin temperature) differences in men with SUD under treatment after detox, depending on the age of OSU and the duration of abstinence. Furthermore, we also explored differences in the circadian rhythm of distal skin temperature between SUD patients and HC participants.

The SUD patients showed a less adaptive sociodemographic pattern than the HC group, with fewer years of education, a greater tendency to remain single and unemployed, consistent with the epidemiological data available on SUD patients (1, 2, 72). This is so regardless of the age of OSU and the duration of abstinence. Given the high impact of the SUD on the functioning of the individuals affected, the data reinforce the need to continue the research on how to improve their prevention and treatment programs.

Both in the total SUD sample and in all the groups of participants, the substances more frequently consumed were cocaine, alcohol, and cannabis, in accordance with both the world and the Spanish data on main substances of clinical diagnosis (2, 72). However, the patients in the OSU ≤ 16 group had a higher frequency of cannabis and hallucinogens consumption than the OSU ≥ 17 group. This may reflect the tendency to use certain substances linked to the life period of onset of consumption (72). Both in the OSU ≤ 16 group and in the SA there is a higher number of patients who need a more intensive treatment (residential) to achieve abstinence compared to the OSU ≥ 17 and MA/LA groups, respectively. These residential programs, in contrast with the ambulatory ones, are addressed to those patients who are experiencing severe drug-related problems (73). In this sense, the OSU ≤ 16 patients presented greater duration of drug use and consumption of more substances, both factors being related to the severity of the addiction (74). These results highlight the evidence that an early age of OSU is related to a more severe clinical symptomatology in adult age (4–6), and are in accordance with the works that use the same cut-off point (7, 8). The better clinical impression of the LA patients compared to the SA group supports the progressive symptomatic recovery as the duration of abstinence increases (75).

Attending to their sleep-wake schedules, there were more morning-type patients in the SUD group, with respect to the normative Spanish data (67). There were no significant differences between the OSU and abstinence groups, and this was more evident in the OSU ≥ 17 group. This tendency to a morning-type was also seen in the advancement of the acrophase of the SUD patients with respect to the HC group. Moreover, the analyses of the parameters of distal skin temperature indicated that, globally, the SUD patients presented a better circadian functioning (higher amplitude, Rayleigh vector, %V, first harmonic power, accumulated power after 12 harmonics, IS, RA, and M5) compared to the HC. These data differ from those reported in previous studies linking eveningness with substance use (37, 40) and those suggesting that circadian rhythmic alteration produced by consumption may last for months after the onset of the abstinence (27–31). This may be explained by the methodological differences, especially by the longer minimum abstinence time (3 months) of the patients in our sample, and also because they do not present active consumption (confirmed by urinalysis), as was also done in Antúnez et al. (76) with SUD patients and with results similar to ours. The therapeutic approach in SUD patients establishes patterns of sleep-wake and regular daily activities in tune with the solar cycle of light-darkness or the morning typology (73), which is considered a protection factor against substance consumption (30, 37). Thus, the patients' correct rhythmic expression could be considered a marker of adherence to treatment and of relapse prevention.

However, some temperature parameters suggest only a partial restitution of the circadian impairment associated to substance consumption. The SUD patients presented high values in minimum temperature, indicative of lower activation and more daytime sleepiness (18, 25), as well as a lower circadianity index or power of the first harmonic, which has been related to an immature circadian system (23). Studying the age of OSU and abstinence groups has contributed to shed light on this issue, showing that this is found only in those patients with lower age of OSU (OSU ≤ 16) and shorter duration of abstinence (SA). Our results indicate that circadian rhythmicity in the SUD group was better when the period of abstinence was longer. The LA group showed higher amplitude, first harmonic power and RA, lower minimum temperature and IV, and delayed TL10 than the SA group, with even better characteristics than the HC group. However, longitudinal works are required that assess the rhythmicity of patients in the long term, once they have completed their treatment.

When the cut-off age of OSU was considered we observed that the OSU ≥ 17 group presented a better circadian distal skin temperature, characterized by higher amplitude, %V, first harmonic power, accumulated power after 12 harmonics and RA. Two hypotheses might explain this observation. First, keeping in mind the implication of the circadian genes in developing an addiction (34, 35), it is possible that the OSU ≤ 16 patients present certain endogenous characteristics previous to the onset of consumption that, added to a worse synchronization of environmental rhythms, could make them more vulnerable to an early onset of substance consumption (77). In this respect, during adolescence an extreme evening-type pattern and the presence of social jet-lag are vulnerability factors for the development of different disorders, including SUD (36, 44). Second, neurotoxicity could underlie the more marked difficulty in restoring the patients' rhythmic expression in spite of following the prescribed treatment. The cut-off age used, based on neurodevelopmental characteristics, discriminates the early phase of maturation of the dopaminergic and endocannabinoid systems (36, 54). Dopamine is the main neuromodulator of the reward system and is involved in the regulation of the clock genes and the circadian rhythms (36, 55). Thus, it is possible that the early consumption of substances affects the correct development of both systems with dysfunctions more perdurable than those produced by a later age of OSU. Unfortunately, the design of our study does not allow us to clarify these issues at the moment.

In this way, the OSU ≤ 16 patients are established as a risk group with a smaller response to interventions, a more severe clinical presentation and greater difficulty in recovering circadian rhythmicity, in comparison with the OSU ≥ 17 patients. Given that a good circadian functioning has been related to better health (9, 10), greater quality of life (49) and better work and academic adjustment (40, 47), these type of patients could benefit from more intense chronobiological strategies, such as the administration of exogenous melatonin, or light-therapy (37). Moreover, since the OSU ≤ 16 patients could have a lower cognitive performance (7, 50–52) and cognitive functions show circadian rhythmicity (13, 15), it could work in favor of their recovery to program more demanding cognitive interventions (such as the cognitive-behavioral) for the time frames when they present more optimal levels of activity.

Our study also has some limitations. A great number of the patients in our sample were polyconsumers, and this made it impossible to tell apart the possible differential effects of each substance on the circadian characteristics, although in our study their effect is relatively under control, since the same main substances had been consumed in the groups. The fact that our sample only consists of men eliminates the influence of gender-related variables, but on the other hand it limits the generalization of the results. The wide range of age in the sample may have also contributed to a type-II error. Moreover, we have not recorded the activity-rest nor the exposition to light, which would have allowed for a more integrated and complete assessment of circadian rhythmicity (20, 78), nor the content and schedule of food/drink intake that may interfere in the rhythm of temperature (11). Finally, we have analyzed cross-sectional data, which does not allow establishing causal relations between variables.

## Conclusions

Our results may have clinical implications. The alterations in the distal skin temperature (a good marker of the circadian system) associated to the SUD may be restored, this being influenced by the duration of abstinence and the age of OSU. Thus, the circadian functioning improves as the time without consumption increases, and this can be considered as an indicator of good adherence and response to treatment (with almost no interference from its modality). In this sense, the distal skin temperature rhythm could be used as a reliable and simple procedure to monitor the patients' response to treatment. Given the relationship between health and circadian rhythms, it would be advisable to do post-intervention follow-ups in order to ensure that the results are maintained. On the other hand, although the age of OSU groups presents similar periods of abstinence and hygienic habits of sleep-wake in the morning-type, the OSU ≤ 16 group appears as the more vulnerable with patients may require from more complex interventions in which it would be beneficial to emphasize the rhythmic aspects. Finally, it would be interesting to include chronobiological assessments in the SUD prevention programs, as well as to boost those chronotherapies addressed to adolescents.

## Availability of data and materials

The raw data supporting the conclusions of this manuscript will be made available by the authors, without undue reservation, to any qualified researcher.

## Author contributions

AA conceived the original idea for the study, sought funding and wrote the protocol. MC and AM-N collected the sample data. MC carried out all the data analyses with input from AA and AM-N. AA, MC, and AM-N participated in the interpretation of the data. AA and MC wrote the manuscript with input from AM-N. All authors have approved the final manuscript.

### Conflict of interest statement

The authors declare that the research was conducted in the absence of any commercial or financial relationships that could be construed as a potential conflict of interest.

## Abbreviations

CGI: Clinical Global Impression questionnaire
CSM: Composite Scale of Morningness
DAST-20: Drug Abuse Screening Test
DSM-IV-TR: Diagnostic and Statistical Manual of Mental Disorders, 4th Edition Text Revised
HC: healthy controls
IS: interdaily stability
IV: intradaily variability
L10: minimum mean temperature in 10 consecutive hours
L2: minimum mean temperature in 2 consecutive hours
LA: long abstinence
M5: maximum mean temperature in 5 consecutive hours
MA: medium abstinence
OSU≤16: age of onset of substance use at age 16 or earlier
OSU≥17: age of onset of substance use at age 17 or later (OSU≥17)
RA: relative amplitude
SA: short abstinence
SCID-I: Structural Clinical Interview for the DSM-IV Axis I Disorders
SPAQ: Seasonal Pattern Assessment Questionnaire
SUD: Substance Use Disorder
TL10: time when the minimum temperature in 10 consecutive hours was reached
TL2: time when the minimum temperature in 2 consecutive hours was reached
TM5: time when the maximum mean temperature in 5 consecutive hours was reached
%V: percentage of variance explained by the cosine wave.
